# The role of PACAP/PAC1R in PTSD: effects on fear extinction via the ventromedial hypothalamus

**DOI:** 10.1192/j.eurpsy.2022.213

**Published:** 2022-09-01

**Authors:** R. Andero

**Affiliations:** Universitat Autònoma de Barcelona, Institute Of Neuroscience, Cerdanyola del Vallès, Spain

**Keywords:** translational, human, mouse, Stress

## Abstract

**Introduction:**

The incidence and severity of posttraumatic stress disorder (PTSD) is higher in women than men because of environmental and biological factors. Specific mechanisms in the PACAP-PAC1R (pituitary adenylate cyclase-activating polypeptide and its type I receptor) system may confer PTSD risk in women. Interestingly, while the PACAP (*ADCYAP1*) - PAC1R (*ADCYAP1R1*) system is expressed highly in the hypothalamus, no relationship has been described for this pathway in the hypothalamus with fear processing or in PTSD.

**Objectives:**

We studied whether the estrous/menstrual cycle at the moment of trauma predicts PTSD and the involvement of the PACAP neurons in the amygdala and hypothalamus during traumatic stress.

**Methods:**

Mice: DREADDs, immunohistochemistry and behavior. Humans: fear-potentiated startle and questionnaires.

**Results:**

Here, we show that acute stress immobilization (IMO) produces fear extinction impairments in female mice. Also, IMO elicits *Adcyap1* and *Adcyap1r1* mRNA upregulation in the hypothalamus, PACAP/c-Fos downregulation in the medial amygdala (MeA), and PACAP/FosB/ΔFosB upregulation in the ventromedial hypothalamus dorsomedial part (VMHdm) after fear extinction. We also found that women with the risk genotype of *ADCYAP1R1* rs2267735 SNP show impaired fear extinction. In mice, DREADD-mediated inhibition of the MeA neurons projecting to the VMHdm during IMO rescues both PACAP upregulation in VMHdm and the fear extinction impairment. We ruled out contributions from inherent hormonal states showing that the menstrual or estrous cycle phase at the moment of trauma does not result in a vulnerable phenotype.

**Conclusions:**

Our data suggest that the PACAP-PAC1R hypothalamic system may be a novel candidate to treat and prevent PTSD symptoms including fear dysregulations.

**Disclosure:**

No significant relationships.

